# Supplementary material for: Wheat Grains as a Sustainable Source of Protein for Health
Source: Nutrients. 2023 Oct 17;15(20):4398. doi: 10.3390/nu15204398 (PMC10609835; doi:10.3390/nu15204398)
Supplement: Supplementary file 1 [file nutrients-15-04398-s001.zip › nutrients-2631287-supplementary.pdf]

Supplementary figures and tables

Table S1. Summary statistics of grain protein concentration in an experiment with 369 European elite wheat genotypes evaluated during 3 years

Trait	Year	Mean	Min.	Max.	Var.	Median	%CV	*s.d*
**GPC (%)**	**2015**	11.85	9.90	14.15	0.404	11.84	5.36	0.64
	**2016**	10.90	9.72	13.12	0.371	10.85	5.58	0.61
	**2017**	11.32	9.52	15.31	0.82	11.29	7.98	0.90
	**BLUEs**	11.37	10.01	13.99	0.274	11.33	4.61	0.52

Min.: Minimun; Max.: Maximun; Var.: Variance; %CV: coefficient of variation; s.d: standard deviation

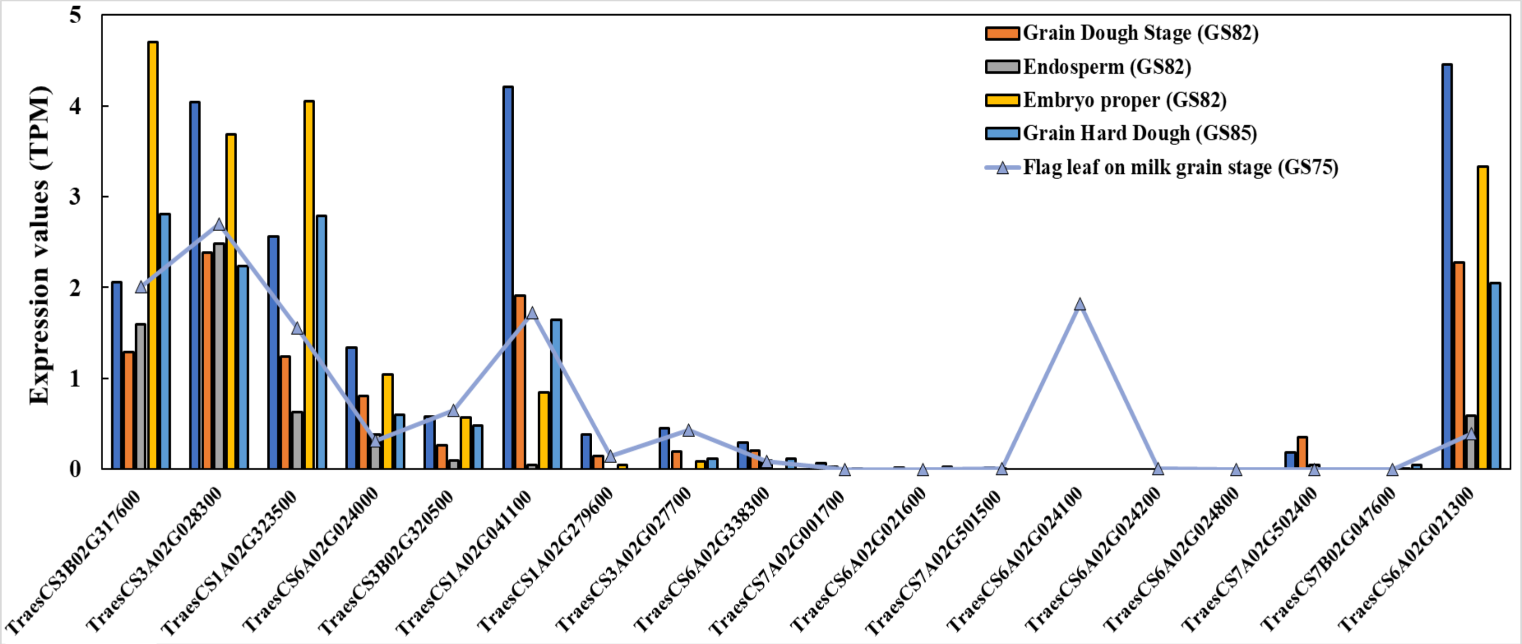

Figure S1. Expression value TPM (Transcripts Per Kilobase Million) of candidate genes in flag leaf (during milk grain stage) and grain development (Milk grain stage, dough stage, endosperm in dough stage, embryo proper in dough stage and grain hard dough)

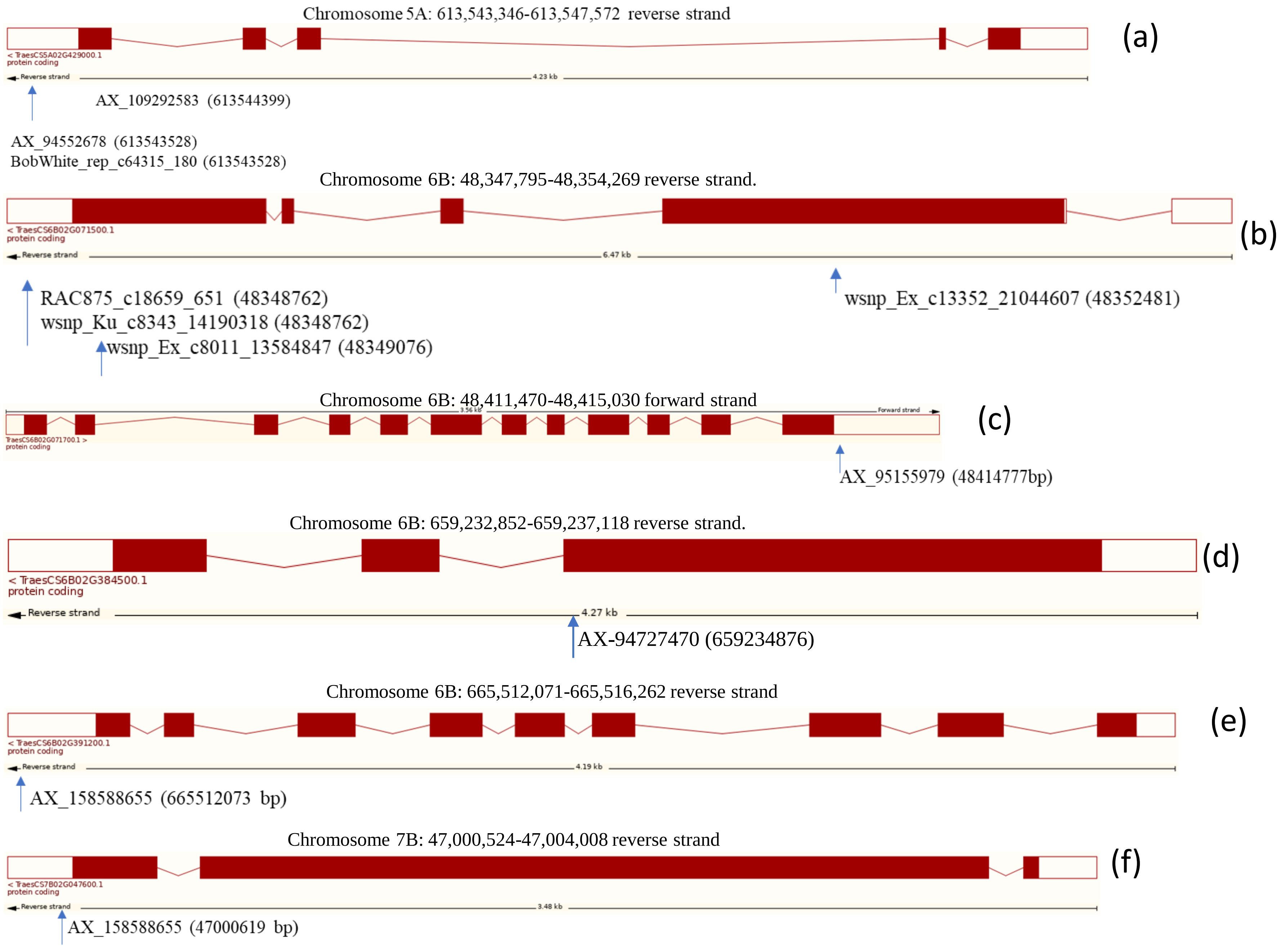

Figure S2. Gene structure and significant QTNs reported (TraesCS5A02G429000 *(a), TraesCS6B02G071500 (b), TraesCS6B02G071700* (c), *TraesCS6B02G384500* (d), *TraesCS6B02G391200* (e), TraesCS7B02G047600 (f) associated with grain protein content in European winter wheat accessions.

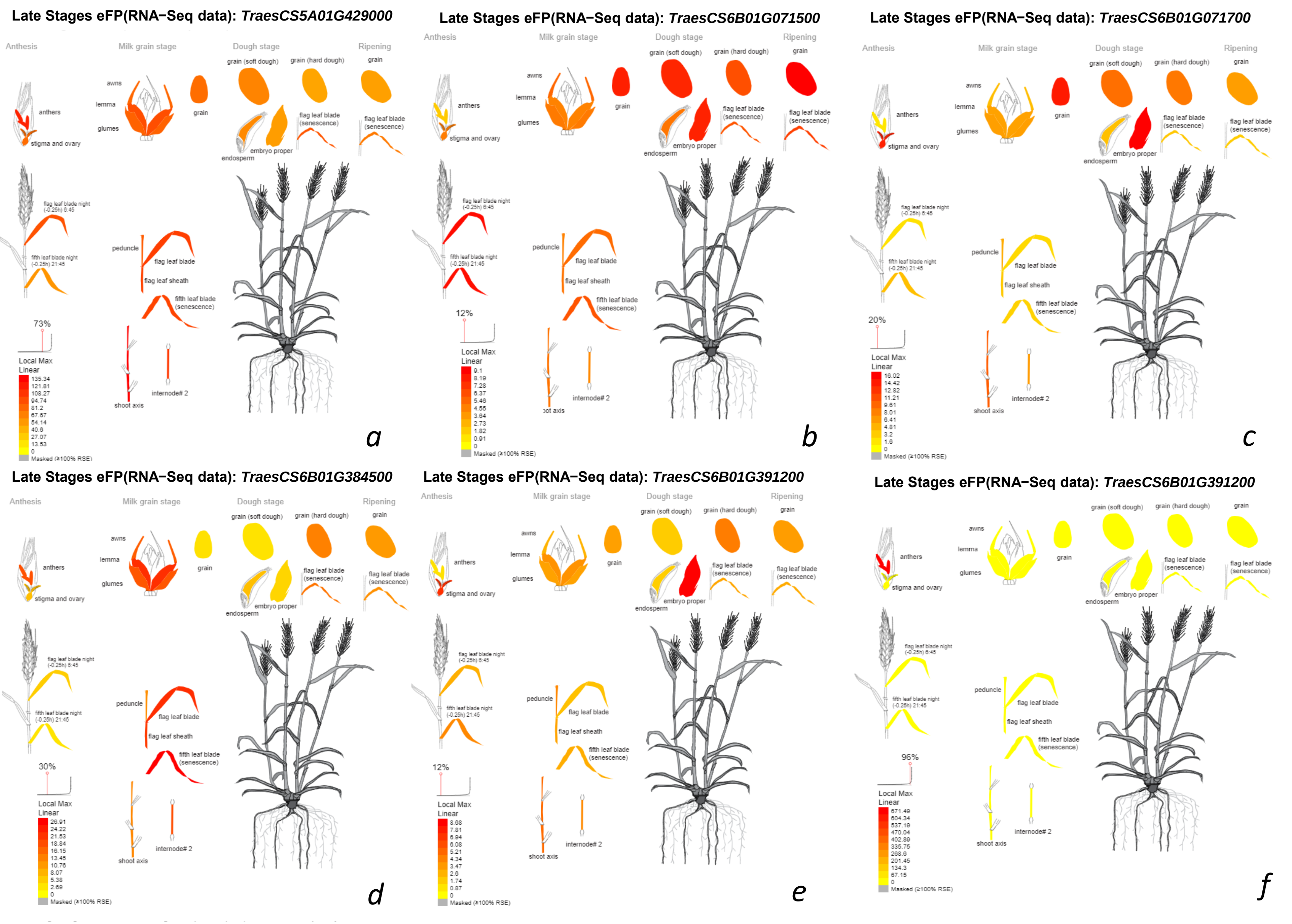

Figure S3. Expression map of the identified gene models (TraesCS5A02G429000 ***(a), TraesCS6B02G071500 (b), TraesCS6B02G071700*** **(c),** ***TraesCS6B02G384500*** **(d),** ***TraesCS6B02G391200*** **(e), TraesCS7B02G047600** ***(f)*** **associated with grain protein content in European winter wheat accessions. Adapted from Waese et al. [72]**

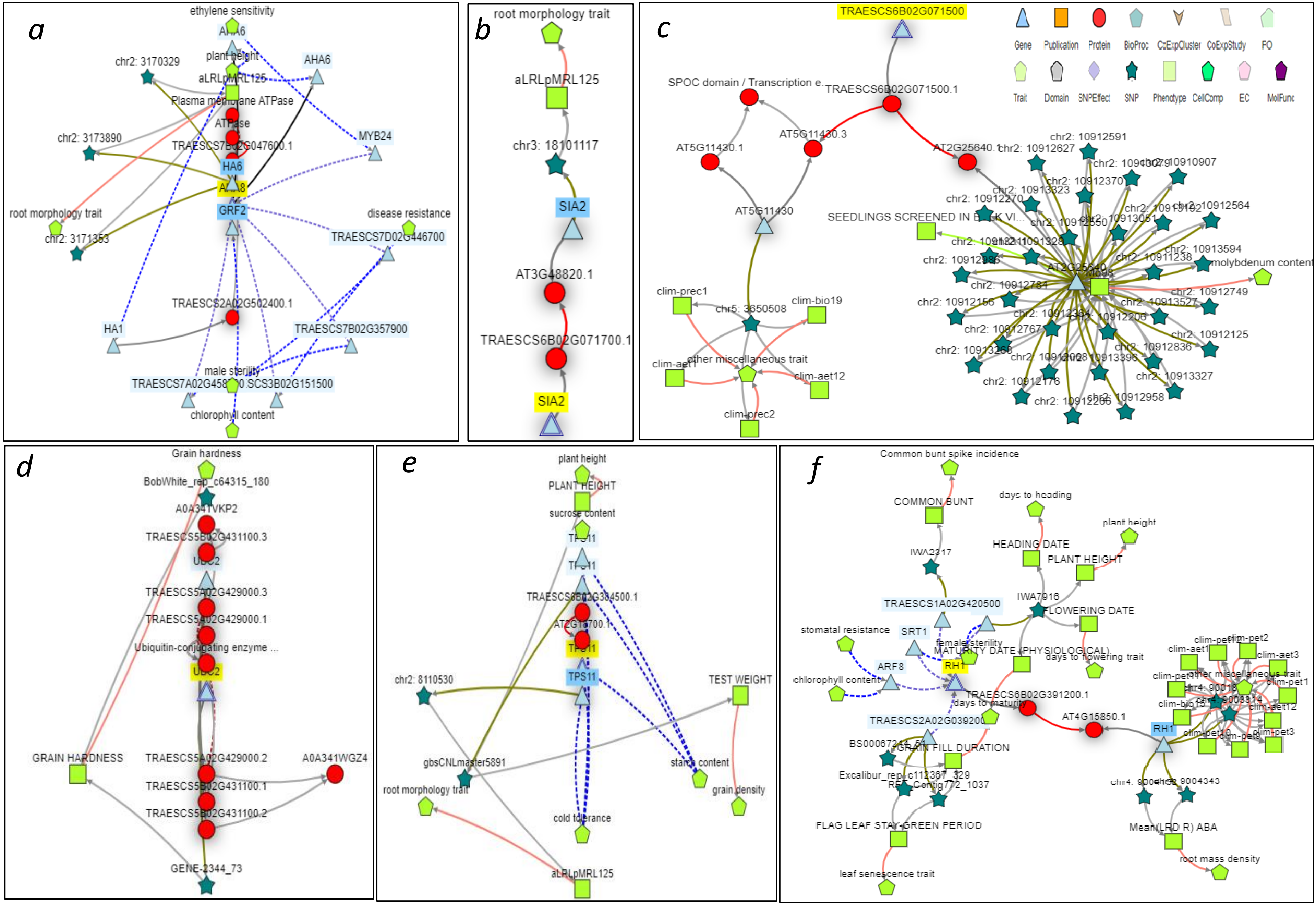

Figure S4. Gene network of the identified gene models ***TraesCS7B02G047600*** **(a.),** ***TraesCS6B02G071700*** **(b.),** ***TraesCS6B02G071500*** **(c.),** ***TraesCS5A02G429000*** **(d.),** ***TraesCS6B02G384500*** **(e.),** ***TraesCS6B02G391200*** **(f.) associated with grain protein content in European winter wheat accessions.**
